# LncRNA ATXN8OS enhances tamoxifen resistance in breast cancer

**DOI:** 10.1515/med-2021-0012

**Published:** 2020-12-15

**Authors:** Hongkai Zhang, Jianni Zhang, Lining Dong, Rong Ma

**Affiliations:** Department of Cell Medicine, International Healthy Cells Rehabilitation Association, Shanghai Liangliang Biotechnology Co., Ltd, No. 876 Taogan Road, Sheshan District 201602, Shanghai, China

**Keywords:** BC, ATXN8OS, miR-16-5p, VASP, TAMR resistance

## Abstract

**Background:**

Tamoxifen (TAMR) resistance remains a massive obstacle for breast cancer (BC) management. The precise parts of long non-coding RNA ataxin 8 opposite strand (ATXN8OS) in BC TAMR resistance have not been defined.

**Methods:**

The levels of ATXN8OS, vasodilator-stimulated phosphoprotein (VASP), and miR-16-5p were assessed by quantitative real-time polymerase chain reaction or western blot. Colony formation and cell viability were analyzed by MTT and colony formation assays, respectively. Targeted interactions among miR-16-5p, ATXN8OS, and VASP were confirmed by dual-luciferase reporter assay. Animal studies were performed to observe the role of ATXN8OS in TAMR sensitivity *in vivo*.

**Results:**

ATXN8OS expression was increased in BC tissues and cells. ATXN8OS depletion promoted BC cell sensitivity to TAMR. ATXN8OS sequestered miR-16-5p by directly binding to miR-16-5p. The promotional effect of ATXN8OS knockdown on BC cell TAMR sensitivity was mediated by miR-16-5p. VASP was a direct target of miR-16-5p, and miR-16-5p overexpression enhanced TAMR sensitivity by VASP. Moreover, ATXN8OS regulated VASP expression by acting as a miR-16-5p sponge. In addition, ATXN8OS knockdown augmented BC TAMR sensitivity *in vivo*.

**Conclusion:**

ATXN8OS knockdown enhanced BC TAMR sensitivity partially through the miR-16-5p/VASP axis, highlighting a potential therapeutic target for improving the clinical benefits of TAMR treatment in BC patients.

## 1 Introduction

Breast cancer (BC) is the most prevalent malignancy and one of the leading causes of cancer-related death among women worldwide, with an estimated 20,88,849 newly diagnosed BC cases and 6,26,679 deaths in 2018 [1]. Tamoxifen (TAMR), an estrogen receptor (ER) antagonist, is the mainstay treatment of BC and is widely used for BC therapy [2]. Despite improved prognosis of BC patients, the development of TAMR resistance remains the major obstacle for BC management [3,4]. Therefore, a better understanding for the potential mechanisms of TAMR resistance is very important to develop new therapeutic targets and improve BC patient prognosis.

Long non-coding RNAs (lncRNAs), a diverse class of >200 nucleotides transcripts, are gaining the attention of researchers in many fields, including cancer [5]. Emerging evidence suggests that dysregulation of lncRNAs is involved in BC tumorigenesis, progression, and TAMR resistance [6,7]. For instance, Xue et al. manifested that lncRNA HOX antisense intergenic RNA (HOTAIR) was upregulated in TAMR-resistant BC tissues and its knockdown repressed cell survival and weakened TAMR-resistant cell growth [4]. Ma and colleagues reported that lncRNA DSCAM-AS1 enhanced TAMR resistance, cell propagation, and hampered apoptosis in BC cells [8]. Ataxin 8 opposite strand (ATXN8OS), an lncRNA with 1,236 bp in length located on chromosome 13q21, was reported to show an aberrant expression in brain diseases [9]. A recent document reported that highly expressed ATXN8OS might be associated with poor prognosis of cervical cancer using a nine-lncRNA signature [10]. Moreover, ATXN8OS was found to be overexpressed in BC tissues and cells, and its depletion repressed BC cell proliferation and invasion, highlighting its role as a tumor promoter in BC [11]. In this study, we focused on the functional role of ATXN8OS in TAMR resistance of BC and underlying mechanisms governing it.

MicroRNAs (miRNAs) are small, endogenous ∼22 nucleotides transcripts that direct gene expression by pairing to the 3′-untranslated region (3′-UTR) of target mRNAs, and thus function as important players in human cancer [12]. Previous researches had reported that miR-16-5p was downregulated in BC tissues, and it exerted a tumor-suppressive role in BC by inhibiting oncogene expression [13,14]. However, the function of miR-16-5p on TAMR resistance in BC remains unclear. Recent studies have reported that lncRNAs regulate TAMR resistance in BC through the networks of competing endogenous RNAs (ceRNAs) via acting as molecular sponges of specific miRNAs [15,16]. The database of starBase 3.0 online software predicted two putative targeted correlations between miR-16-5p and ATXN8OS or vasodilator-stimulated phosphoprotein (VASP), eliciting a potential network of ATXN8OS/miR-16-5p/VASP axis. In the present study, we aimed to explore the detailed role and underlying mechanisms of ATXN8OS on TAMR resistance in BC. By combining *in vitro* and *in vivo* experiments, our study suggested that the knockdown of ATXN8OS augmented TAMR sensitivity in BC cells possibly through miR-16-5p/VASP axis.

## 2 Materials and methods

### 2.1 Clinical samples and ethical statement

In this study, 44 tissue samples were collected, including 22 BC tissues and 22 adjacent normal breast tissues, from International Healthy Cells Rehabilitation Association, Shanghai Liangliang Biotechnology Co., Ltd. Fresh samples were stored at −80°C until RNA extraction. The protocol of this study was approved by the Ethics Committee of International Healthy Cells Rehabilitation Association, Shanghai Liangliang Biotechnology Co., Ltd, and written informed consent was obtained from all the participating subjects.


**Ethics approval and consent to participate:** The present study was approved by the ethical review committee of International Healthy Cells Rehabilitation Association, Shanghai Liangliang Biotechnology Co., Ltd.
**Patient consent for publication:** Not applicable.

### 2.2 Cell culture, treatment, and transfection

Human normal mammary epithelial cell line MCF-10A (ATCC^®^CRL-10317), and two BC cell lines MCF-7 (ATCC^®^HTB-22^TM^) and BT-549 (ATCC^®^HTB-122) were bought from the American Type Culture Collection (ATCC, Manassas, VA, USA). All the cells were maintained at 37°C, 5% CO_2_ in RPMI-1640 medium (ATCC), plus 10% fetal bovine serum (FBS, ATCC) and 1% penicillin/streptomycin (Gibco, Cergy Pontoise, France). For TAMR exposure, BC cells were treated with various concentrations (0.5, 1, 2, 4, 8, 16, 32, 64, and 128 µm) or 10 µm of TAMR (Gibco) for 48 h.

For the knockdown of ATXN8OS, cells were transfected with synthetic small interfering RNA (siRNA) against ATXN8OS (si-ATXN8OS, GenePharma, Shanghai, China), and nontarget siRNA (si-NC, GenePharma) was used as the negative control. For miR-16-5p overexpression or depletion, modified miR-16-5p mimic (GenePharma), inhibitor of miR-16-5p (anti-miR-16-5p, GenePharma), or corresponding negative control (miR-NC mimic or anti-miR-NC, GenePharma) was introduced into cells. VASP upregulation was carried out using VASP overexpression plasmid (the full sequence of VASP was cloned into pcDNA3.1, GenePharma), with nontarget pcDNA3.1 plasmid as the negative control (vector, GenePharma). The Lipofectamine 3000 reagent (Invitrogen, Cergy Pontoise, France) was used for each transfection following the protocols of manufacturers.

### 2.3 Determination of IC_50_ value for TAMR

A basic colorimetric 3-(4,5-dimethylthiazol-2-yl)-2,5-diphenyltetrazolium bromide (MTT) assay was implemented to determine the IC_50_ value for TAMR. Cells (1.0 × 10^5^ each well) seeded in 96-well plates were transfected with the indicated oligonucleotides or plasmids and then were exposed to various concentrations (0.5, 1, 2, 4, 8, 16, 32, 64, and 128 µm) of TAMR. After 48 h exposure, MTT solution (Sigma-Aldrich) was used at a final concentration of 0.5 mg/mL per well, followed by the incubation for 2 h at 37°C. Then, media was removed and dimethyl sulfoxide (DMSO, 200 µL each well) was added into plates to dissolve formazan crystals. The quantity of formazan was proportional to the number of viable cells, which could be measured using a microplate reader (Thermo Fisher Scientific, Braunschweig, Germany) at the wavelength of 490 nm.

### 2.4 Cell apoptosis assay

Flow cytometry was performed to evaluate cell apoptosis using the Annexin V-FITC/PI apoptosis kit (Thermo Fisher Scientific). Briefly, cells were transfected with the indicated oligonucleotides or plasmids and then were treated with 10 µm of TAMR (Gibco) for 48 h. After that, cells were stained with 5 µL of Annexin V-FITC and 10 µL of PI for 10 min in the dark. The flow cytometer (BD Biosciences, San Jose, CA, USA) was finally used for the assessment of cell apoptotic rate.

### 2.5 Western blot

Cells and tissues were homogenized in RIPA lysis buffer (Beyotime, Shanghai, China) containing protease inhibitor cocktail (Sigma-Aldrich). After centrifugation at 13,000 g for 20 min, the supernatant was collected and quantified using a BCA protein assay kit (Thermo Fisher Scientific). Total protein (50 µg) was subjected to electrophoresis on a 10% SDS polyacrylamide gel and then transferred to polyvinylidene difluoride (PVDF) membranes (Millipore, Molsheim, France). After being blocked in 5% non-fat powdered milk, the membranes were probed with primary antibodies at the indicated dilution, followed by the incubation with HRP-conjugated anti-mouse or anti-rabbit IgG secondary antibodies (Abcam, Cambridge, UK). Protein bands were detected using SuperSignal West Pico PLUS chemiluminescent substrate (Thermo Fisher Scientific) and analyzed by GeneTools software (Syngene, Cambridge, UK). The following antibodies were used: anti-Beclin1 (Abcam; dilution 1:1,000), anti-light chain 3 I (anti-LC3 I, Cell Signaling Technology, Danvers, MA, USA; dilution 1:1,000), anti-LC3 II (Cell Signaling Technology; dilution 1:1,000), anti-Bcl-2 (Abcam; dilution 1:1,000), anti-Bax (Abcam; dilution 1:1,000), and anti-VASP (Abcam; dilution 1:3,000). Expression of glyceraldehyde-3-phosphate dehydrogenase was used as a loading control.

### 2.6 Quantitative real-time polymerase chain reaction

Total RNA was prepared using SV total RNA isolation system (Promega, Madison, WI, USA) referring to the manufacturer’s protocols. The quantity and quality of RNA extracts were analyzed by the Agilent 2100 Bioanalyzer (Agilent Technologies, Waldbronn, Germany). Complementary DNA (cDNA) was synthesized using the Maxima H Minus First Strand cDNA synthesis kit (Thermo Fisher Scientific) with random hexamer primers for ATXN8OS and VASP mRNA and TaqMan MicroRNA reverse transcription kit (Applied Biosystems, Bleiswijk, The Netherlands) with target-specific stem-loop primers for miR-16-5p, referring to the instructions of manufacturers. The expression levels of ATXN8OS, VASP mRNA, and miR-16-5p were determined by quantitative real-time polymerase chain reaction (qRT-PCR) using SYBR^®^ Premix Ex Taq^TM^ reagent (Takara, Dalian, China), with β-actin or U6 snRNA as the internal control. Primers sequences (5′–3′) for qRT-PCR were as follows: ATXN8OS: CATTACTGTGTAGCAATAC (sense) and AATTCAAGCCATTAACCT (antisense); VASP: CTGGGAGAAGAACAGCACAACC (sense) and AGGTCCGAGTATCACTGGAGC (antisense); β-actin: GATGGAAATCGTCAGAGGCT (sense) and TGGCACTTAGTTGGAAATGC (antisense); miR-16-5p: CGCGCTAGCAGCACGTAAAT (sense) and GTGCAGGGTCCGAGGT (antisense); U6: GCTTCGGCAGCACATATACTAAAAT (sense) and CGCTTCACGAATTTGCGTGTCAT (antisense).

### 2.7 Bioinformatics and dual-luciferase reporter assay

Bioinformatic analyses for the directly interacted miRNAs of ATXN8OS and the molecular targets of miR-16-5p were performed using starBase 3.0 online software at http://starbase.sysu.edu.cn/. The partial sequences of ATXN8OS and VASP 3′-UTR harboring the wild-type target sequence for miR-16-5p were cloned into the pmirGLO vector (Promega), respectively, to construct ATXN8OS and VASP 3′-UTR wild-type luciferase reporter plasmids (ATXN8OS WT and VASP 3′-UTR WT). Site-directed mutants (ATXN8OS MUT and VASP 3′-UTR MUT) were generated using the GENEART Site-Directed Mutagenesis System (Thermo Fisher Scientific) following the producer’s guidance. The reporter constructs were cotransfected into MCF-7 and BT-549 cells together with miR-16-5p mimic or miR-NC mimic. The luciferase activities were measured after 48 h transfection using the dual-luciferase reporter assay system (Promega).

### 2.8 Lentiviral vector transduction

Lentiviral vectors containing short hairpin RNA (shRNA) against ATXN8OS (sh-ATXN8OS) or nontarget control shRNA (sh-NC) were obtained from Genechem (Shanghai, China). MCF-7 cells were transduced by sh-ATXN8OS or sh-NC with various multiplicities of infection in media containing 8 µg/mL of polybrene. Twenty-four hours after transduction, the cells with positive infection were selected using puromycin at a final concentration of 1 µg/mL.

### 2.9 Xenograft model assay

To implement *in vivo* assays, female 6-week-old BALB/c nude mice (Henan Research Center of Laboratory Animal, Zhengzhou, China) were used in the study, after obtaining approval from Animal Ethical Committee of International Healthy Cells Rehabilitation Association, Shanghai Liangliang Biotechnology Co., Ltd. About 5.0 × 10^6^ MCF-7 cells stably transduced with sh-NC or sh-ATXN8OS were subcutaneously inoculated into the left flank of nude mice, followed by the administration of TAMR (50 mg/kg) every 5 days after 5 days of implantation (*n* = 9 each group). Tumor volume was measured with a caliper every 1 week and calculated with the formula: length (*L*) × width (*W*)^2^ = mm^3^. Thirty-five days after injection, all mice were killed to remove the tumor tissues. Animal experiments were carried out following the Guide for the Care and Use of Laboratory Animals of International Healthy Cells Rehabilitation Association, Shanghai Liangliang Biotechnology Co., Ltd.

### 2.10 Statistical analysis

Data analysis and statistical calculation were conducted by SPSS v.22 software (SPSS Inc., Chicago, IL, USA) using a Student’s *t*-test (two-tailed) and one-way analysis of variance (ANOVA), followed by post hoc analysis. All the results were expressed as mean ± standard error of the mean (SEM) and a *p*-value <0.05 was considered statistically significant.

## 3 Results

### 3.1 ATXN8OS was upregulated in BC tissues and cells

**Figure 1.**
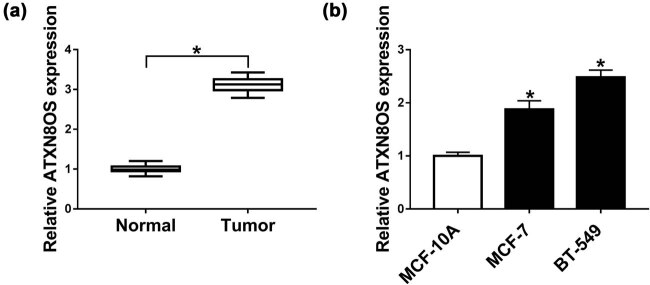
ATXN8OS was highly expressed in BC tissues and cells. ATXN8OS expression by qRT-PCR in 22 pairs of BC tissues and adjacent normal breast tissues (a) and MCF-10A, MCF-7, and BT-549 cells (b). *P < 0.05.

First, we determined the expression of ATXN8OS in BC tissues and cells. As shown by qRT-PCR, ATXN8OS expression was higher in BC tissues than that in normal breast tissues (Figure 1a). In parallel, in comparison to the normal control, ATXN8OS expression was significantly increased in BC cells (Figure 1b).

### 3.2 ATXN8OS knockdown augmented TAMR sensitivity of BC cells

**Figure 2:**
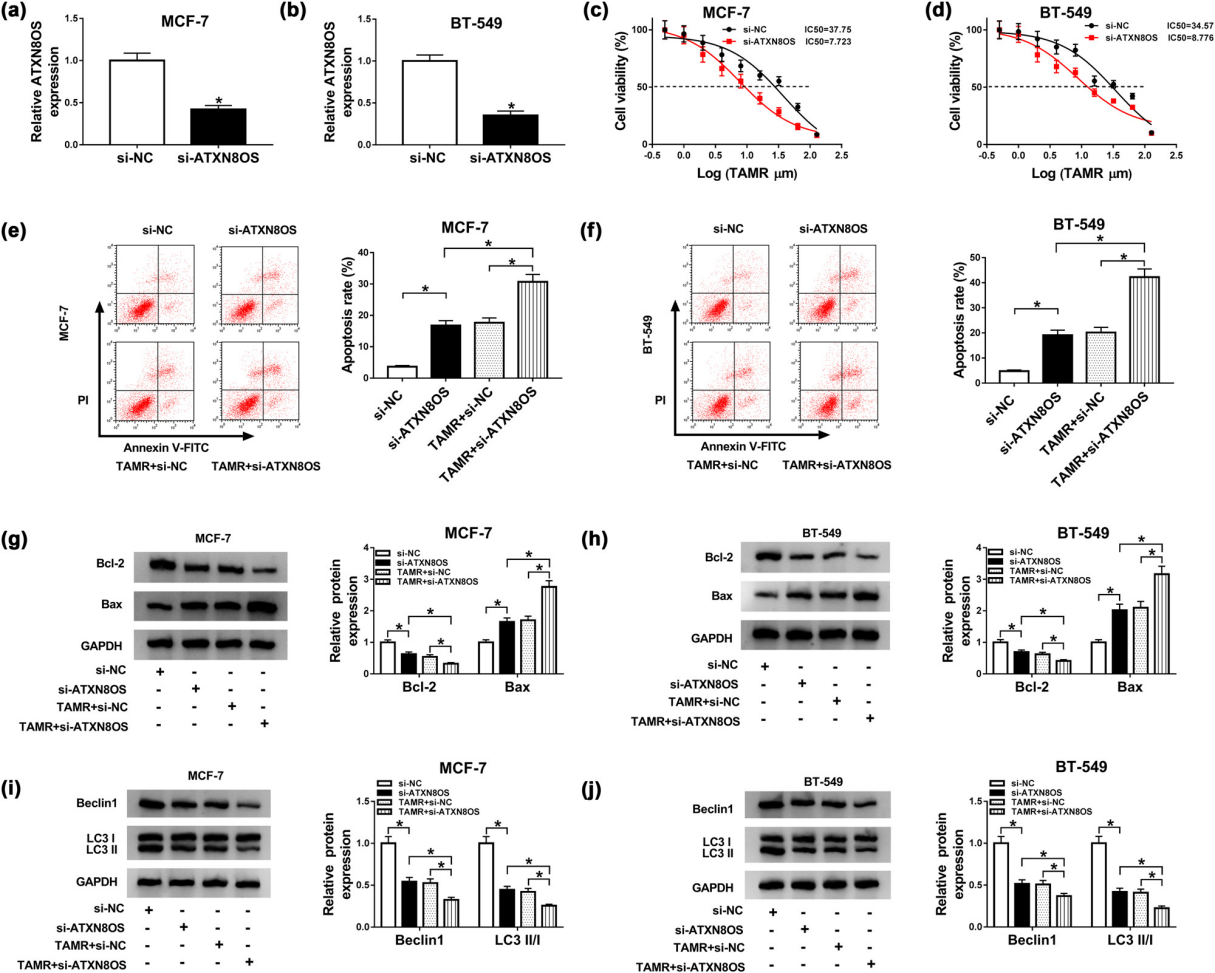
ATXN8OS depletion enhanced TAMR sensitivity in BC cells. (a and b) MCF-7 and BT-549 cells were transfected with si-NC or si-ATXN8OS, followed by the determination of ATXN8OS expression by qRT-PCR 48 h post-transfection. (c and d) MCF-7 and BT-549 cells were transfected with si-NC or si-ATXN8OS and then were exposed to various concentrations (0.5, 1, 2, 4, 8, 16, 32, 64, and 128 µm) of TAMR for 48 h, followed by the measurement of cell viability using MTT assay. MCF-7 and BT-549 cells were transfected with si-NC or si-ATXN8OS and then were treated with 10 µm of TAMR for 48 h, followed by the determination of cell apoptosis by flow cytometry (e and f), Bcl-2 and Bax levels by western blot (g and h), and the levels of Beclin 1, LC3 I, and LC3 II by western blot (i and j). *P < 0.05.

To explore the role of ATXN8OS in BC TAMR resistance, loss-of-function experiments were performed by si-ATXN8OS. Transient introduction of si-ATXN8OS, but not a scrambled control sequence, induced a prominent reduction in ATXN8OS expression (about 58% reduction in MCF-7 cells and 65% reduction in BT-549 cells; Figure 2a and b). MTT assay revealed that ATXN8OS knockdown resulted in decreased IC_50_ value for TAMR in the two BC cells (Figure 2c and d). The data of flow cytometry and western blot showed that ATXN8OS depletion led to a significant promotion in cell apoptosis (Figure 2e and f), a distinct reduction in Bcl-2 expression, and a clear enhancement in Bax level (Figure 2g and h) with or without TAMR treatment. Moreover, the knockdown of ATXN8OS triggered the striking repression in Beclin 1 expression and LC3 II/I ratio (Figure 2i and j) in the two BC cells when treatment with or without TAMR, suggesting the suppressive role of ATXN8OS knockdown in autophagy. More interestingly, simultaneous ATXN8OS silencing and TAMR treatment resulted in a more significant promotion in cell apoptosis (Figure 2e–h) and a more clear repression in cell autophagy (Figure 2i and j).

### 3.3 ATXN8OS acted as a molecular sponge of miR-16-5p to sequester miR-16-5p

**Figure 3:**
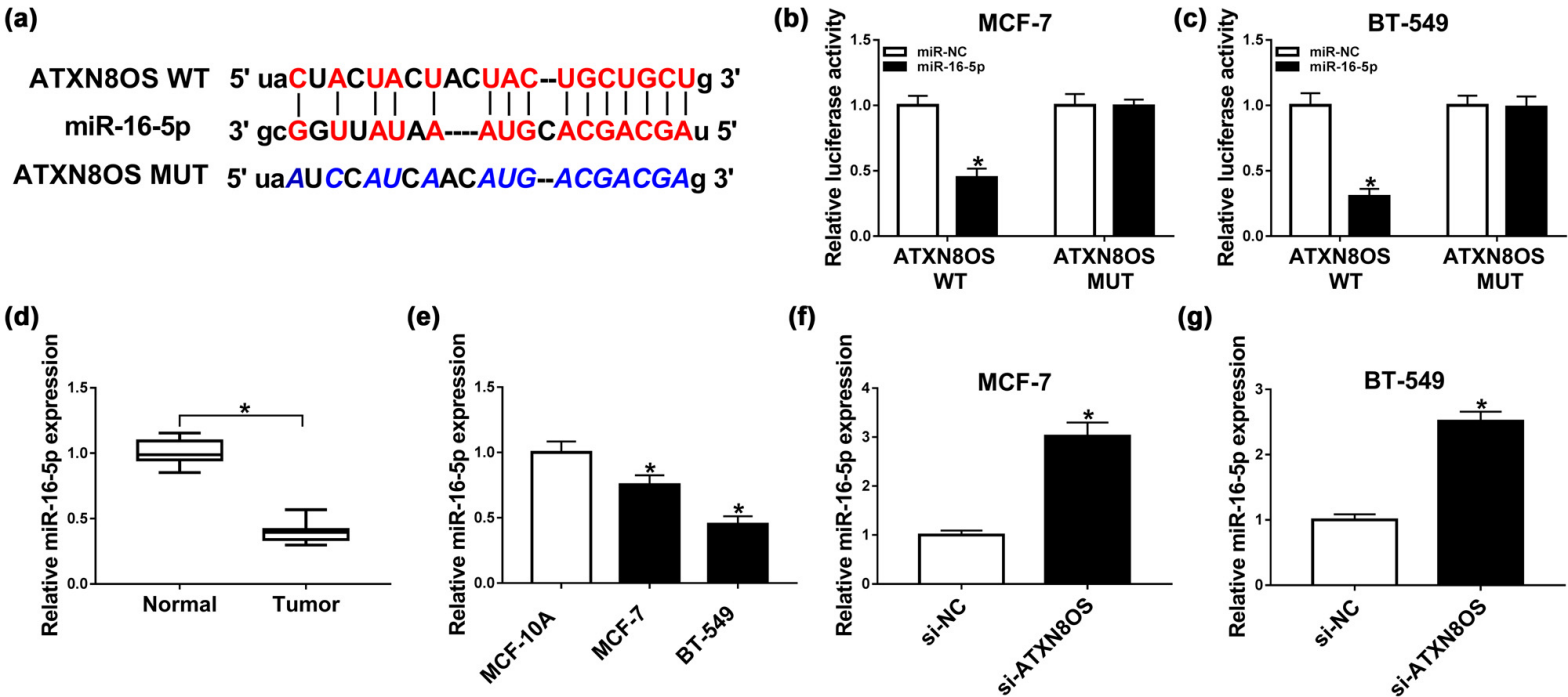
ATXN8OS sequestered miR-16-5p by sponging miR-16-5p. (a) Schematic of the putative miR-16-5p-binding site in ATXN8OS by starBase 3.0 software and mutated miR-16-5p-binding site. (b and c) The luciferase activity in MCF-7 and BT-549 cells cotransfected with ATXN8OS luciferase reporter construct harboring the wild-type target sequence for miR-16-5p (ATXN8OS WT) or its mutant in seed sequence (ATXN8OS MUT) and miR-16-5p mimic or miR-NC mimic. The expression of miR-16-5p by qRT-PCR in 22 pairs of BC tissues and adjacent normal breast tissues (d), and MCF-10A, MCF-7, and BT-549 cells (e). (f and g) The expression of miR-16-5p in MCF-7 and BT-549 cells transfected with si-NC or si-ATXN8OS. *P < 0.05.

To further explore the molecular mechanism by which ATXN8OS influenced BC TAMR resistance, we carried out a detailed analysis for the directly interacted miRNAs of ATXN8OS. Using starBase 3.0 software, 10 targeted miRNAs were predicted (Supplement Figure S1a). qRT-PCR data showed that miR-16-5p was the most significantly upregulated miRNA when ATXN8OS silencing in MCF7 and BT-549 cells (Supplement Figure S1b and c). Therefore, we selected miR-16-5p for further analyses. To confirm the targeted interaction between ATXN8OS and miR-16-5p, we cloned ATXN8OS segment harboring the putative miR-16-5p-binding sequence into a luciferase vector and mutated the target region (Figure 3a). With the wild-type reporter construct and miR-16-5p upregulation caused a significant reduction in luciferase activity (Figure 3b and c). When the miR-16-5p-binding site was mutated, little reduction in luciferase activity was observed in the presence of miR-16-5p mimic (Figure 3b and c). Our data also showed that miR-16-5p was prominently downregulated in BC tissues and cells, as compared to their counterparts (Figure 3d and e). After that, we observed whether ATXN8OS modulated miR-16-5p expression in the two BC cells. As expected, in contrast to negative group, transfection of si-ATXN8OS resulted in increased miR-16-5p expression (Figure 3f and g). Together, these results strongly pointed a role of ATXN8OS as a molecular sponge of miR-16-5p.

### 3.4 The promotional effect of ATXN8OS knockdown on TAMR sensitivity was abated by restored expression of miR-16-5p in BC cells

**Figure 4:**
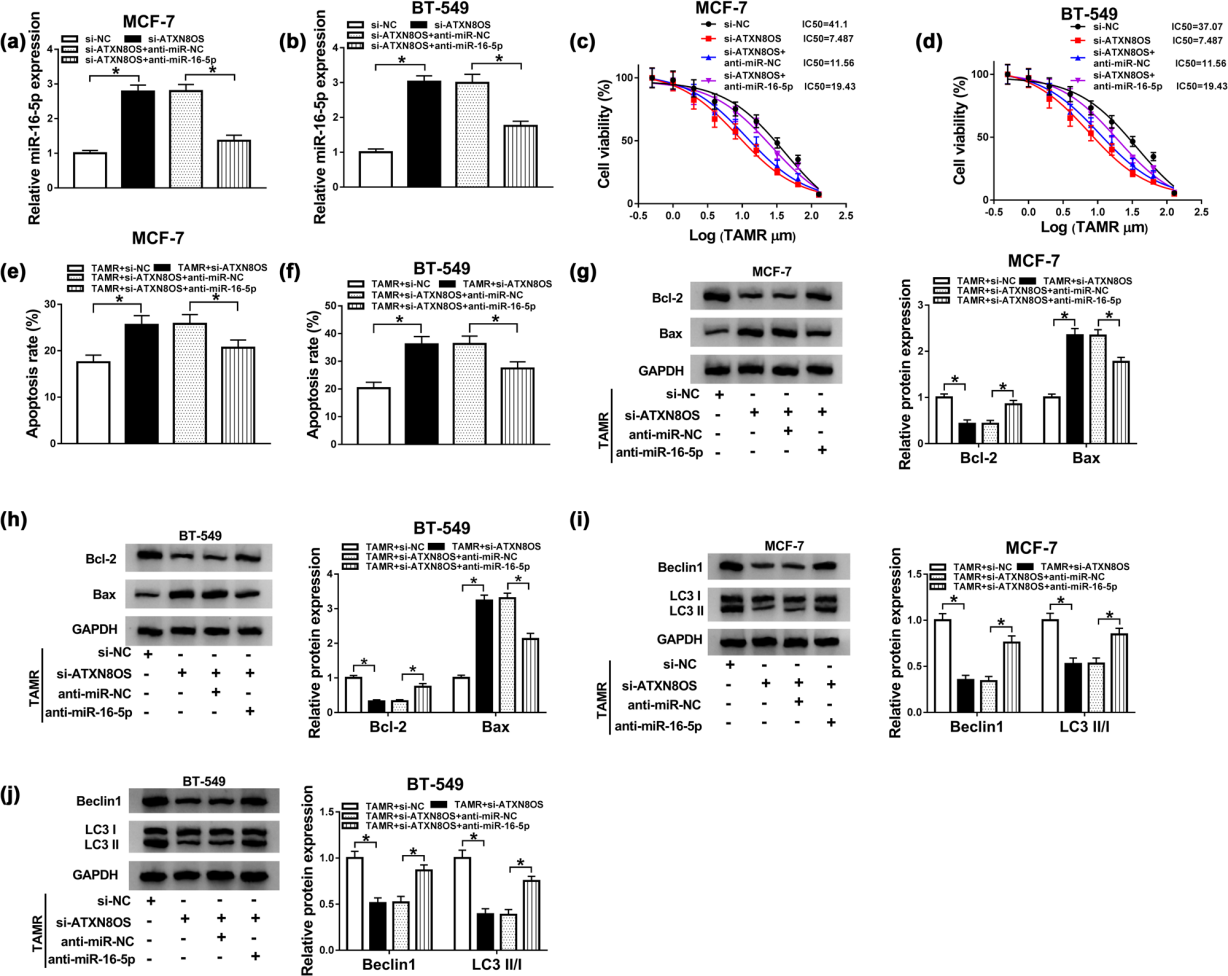
The knockdown of ATXN8OS enhanced TAMR sensitivity by upregulating miR-16-5p. MCF-7 and BT-549 cells were transfected with si-NC, si-ATXN8OS, si-ATXN8OS + anti-miR-NC, and si-ATXN8OS + anti-miR-16-5p before TAMR treatment with the indicated concentrations, followed by the measurement of miR-16-5p expression by qRT-PCR (a and b), the IC_50_ value for TAMR by MTT assay (c and d), cell apoptosis by flow cytometry (e and f), Bcl-2 and Bax levels by western blot (g and h), and the levels of Beclin 1, LC3 I and LC3 II by western blot (i and j). *P < 0.05.

Then, we further investigated whether ATXN8OS exerted its regulatory effect on BC TAMR resistance by miR-16-5p. As shown by qRT-PCR, si-ATXN8OS-mediated miR-16-5p increase was significantly abolished by cotransfection of anti-miR-16-5p in the two BC cells (Figure 4a and b). Subsequent experiments’ results revealed that in comparison to the negative group, the reduced effect of ATXN8OS depletion on IC_50_ value was markedly reversed by miR-16-5p expression restoration (Figure 4c and d). Moreover, si-ATXN8OS-mediated pro-apoptosis (Figure 4e–h) and anti-autophagy (Figure 4i and j) effects under TAMR treatment were prominently abrogated by restored expression of miR-16-5p.

### 3.5 VASP was directly targeted and inhibited by miR-16-5p

**Figure 5:**
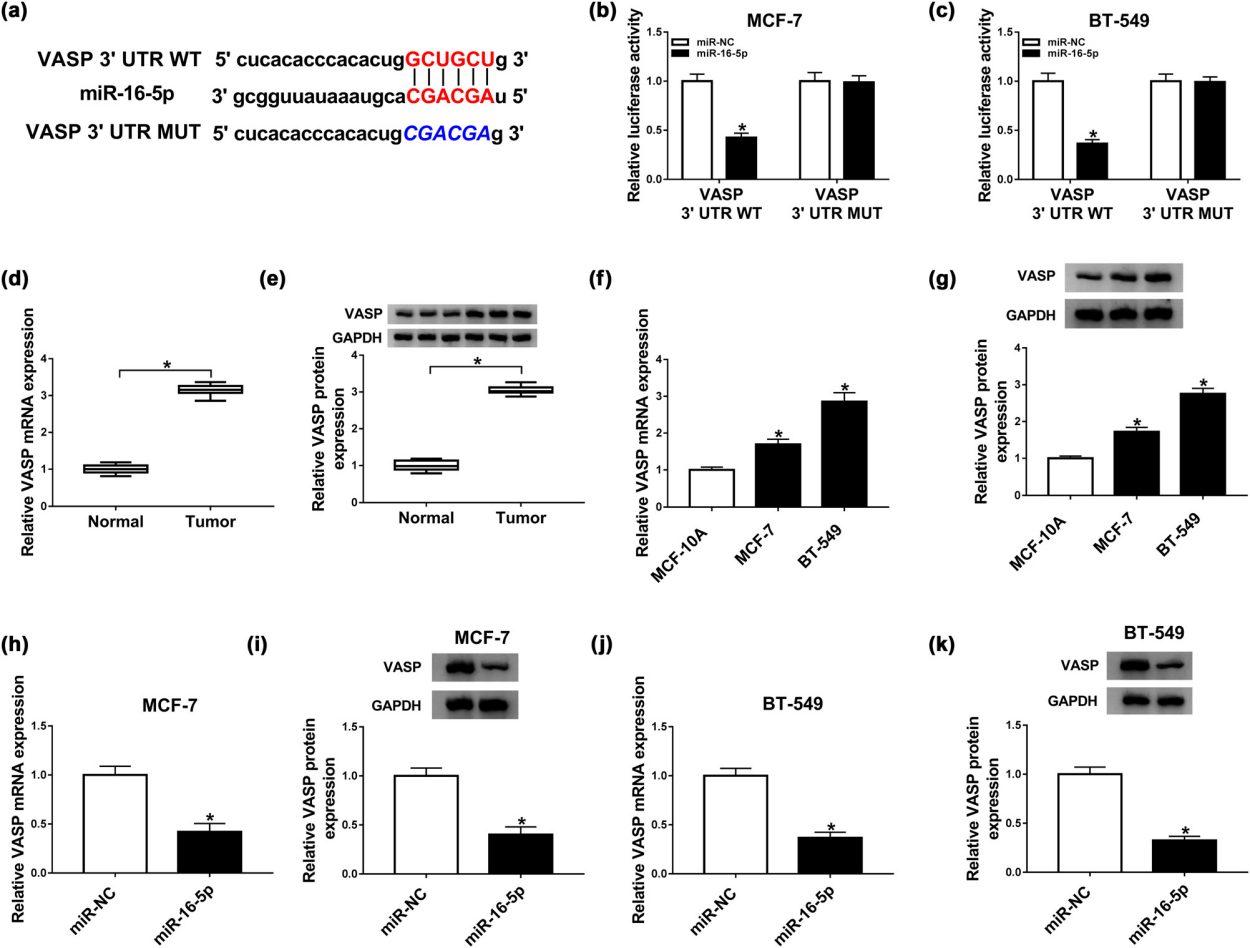
VASP was a direct target of miR-16-5p. (a) Schematic of sequence complementarity between miR-16-5p and VASP 3′-UTR and the mutant in complementary sequence. (b and c) The luciferase activity in MCF-7 and BT-549 cells cotransfected with VASP 3′-UTR wild-type reporter (VASP 3′-UTR WT) or site-directed mutant of the target sequence (VASP 3′-UTR MUT) and miR-NC mimic or miR-16-5p mimic. The mRNA and protein levels of VASP in 22 pairs of BC tissues and adjacent normal breast tissues (d and e), MCF-10A, MCF-7, and BT-549 cells (f and g), MCF-7 (h and i), and BT-549 (j and k) cells transfected with miR-NC mimic or miR-16-5p mimic. *P < 0.05.

To further understand the role of miR-16-5p in BC TAMR resistance, we used starBase 3.0 software to help identify its molecular targets in humans. Among the thousands of candidates, VEGFA, VASP, E2F3, and HDGF were of interest in the present study because their expression levels had been found to be upregulated in BC [17,18,19,20]. Western blot data validated the overexpression of the four targets in BC cells compared with MCF-10A cells (Supplement Figure 1d). Moreover, VASP expression the most significantly down-regulated in the miR-16-5p-overexpressing BC cells (Supplement Figure 1e). To verify the targeted relationship between VASP and miR-16-5p, we performed dual-luciferase reporter assay using VASP 3′-UTR reporter (Figure 5a). The cotransfection of VASP 3′-UTR wild-type reporter and miR-16-5p mimic into the two BC cells produced lower luciferase activity than in cells cotransfected with miR-NC mimic (Figure 5b and c). However, site-directed mutant of the target sequence strikingly abolished the effect of miR-16-5p on reporter gene expression under the same conditions (Figure 5b and c). Our data also revealed that the mRNA and protein levels of VASP were significantly upregulated in BC tissues (Figure 5d and e) and cells (Figure 5f and g) compared with their counterparts. Moreover, qRT-PCR and western blot analyses showed that in contrast to miR-NC mimic group, the mRNA and protein levels of VASP were remarkably reduced by miR-16-5p overexpression in both MCF-7 (Figure 5h and i) and BT-549 (Figure 5j and k) cells.

### 3.6 VASP mediated the promotional effect of miR-16-5p overexpression on TAMR sensitivity in BC cells

**Figure 6:**
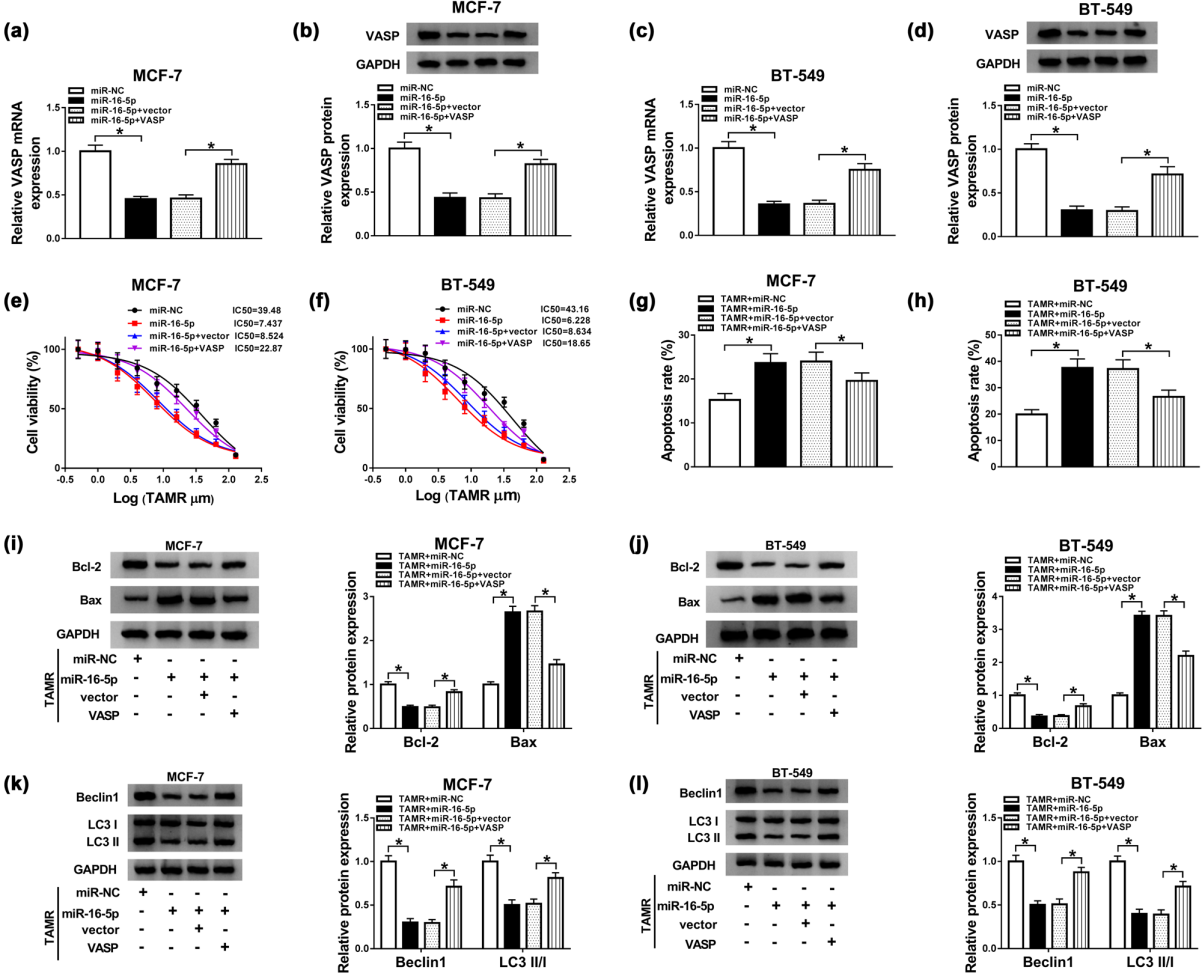
The overexpression of miR-16-5p promoted TAMR sensitivity by VASP. MCF-7 and BT-549 cells were transfected with miR-NC mimic, miR-16-5p mimic, miR-16-5p mimic + vector, or miR-16-5p mimic + VASP (VASP overexpression plasmid) before TAMR treatment with the indicated concentrations, followed by the determination of VASP mRNA and protein levels (a–d), the IC_50_ value for TAMR by MTT assay (e and f), cell apoptosis by flow cytometry (g and h), Bcl-2 and Bax levels by western blot (i and j), and the levels of Beclin 1, LC3 I and LC3 II by western blot (k and l). *P < 0.05.

To provide further mechanistic insights into the link between miR-16-5p and VASP on BC TAMR resistance, MCF-7 and BT-549 cells were transfected with miR-16-5p mimic alone or together with VASP overexpression plasmid. Our data showed that VASP level was significantly reduced by miR-16-5p overexpression in the two BC cells using qRT-PCR and western blot assays (Figure 6a–d). Moreover, the decreased effect of miR-16-5p overexpression on VASP level was highly abated by cotransfection of VASP overexpression plasmid (Figure 6a–d). In contrast to miR-NC mimic group, miR-16-5p overexpression led to a significant reduction in IC_50_ value for TAMR (Figure 6e and f) and a distinct enhancement in cell apoptosis (Figure 6g–-j), as well as a clear suppression of cell autophagy (Figures 6k and l) under TMAR treatment. These data together established a promotional effect of miR-16-5p overexpression on TAMR sensitivity in the two BC cells. However, this effect was significantly reversed by VASP expression restoration in both MCF-7 and BT-549 cells (Figure 6e–l) under TAMR exposure.

### 3.7 ATXN8OS regulated VASP expression by miR-16-5p and VASP was a functional target of ATXN8OS in regulating BC cell autophagy

**Figure 7:**
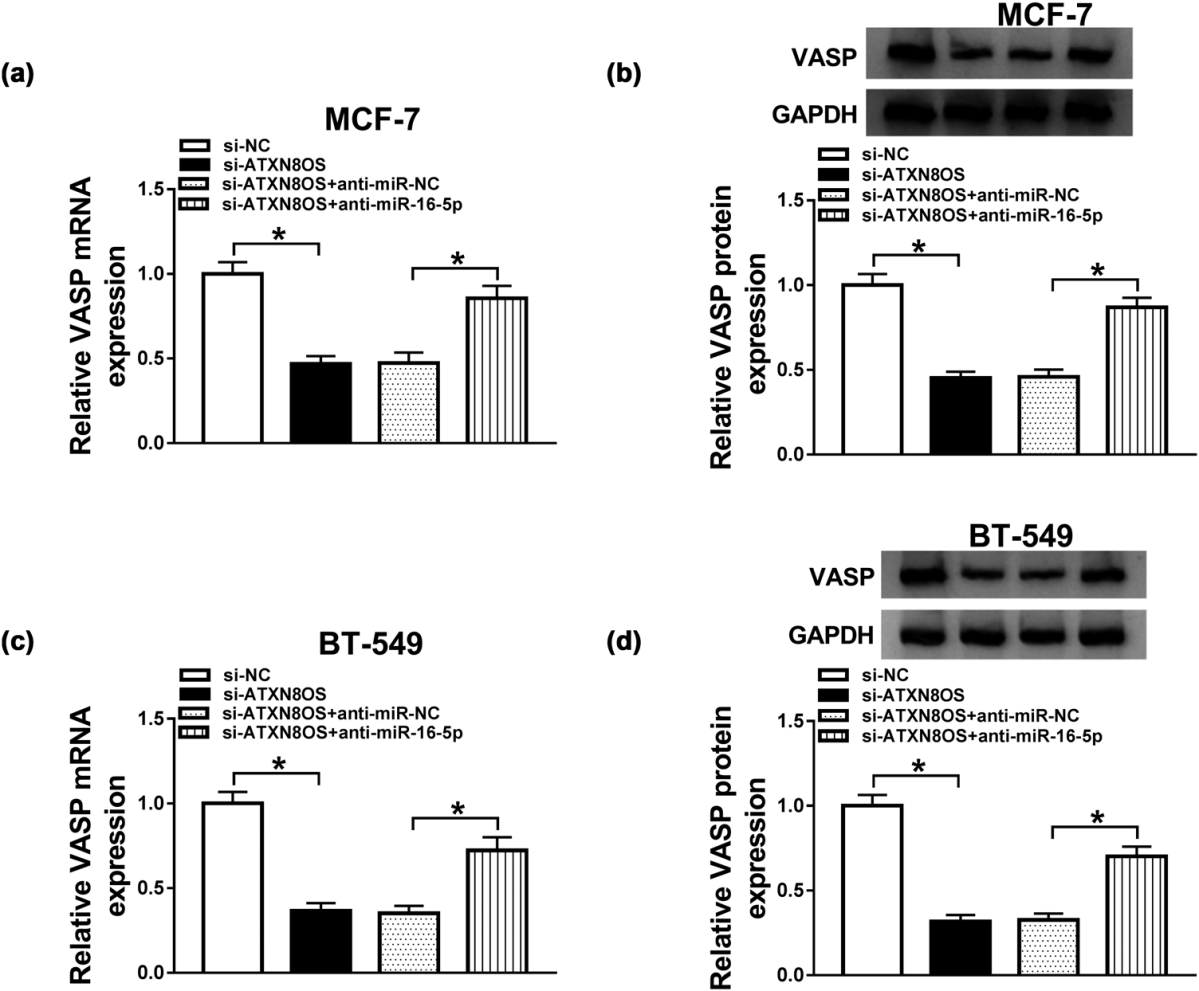
ATXN8OS acted a ceRNA of miR-16-5p to regulate VASP expression. MCF-7 (a and b) and BT-549 (c and d) cells were transfected with si-NC, si-ATXN8OS, si-ATXN8OS + anti-miR-NC, or si-ATXN8OS + anti-miR-16-5p, followed by the evaluation of VASP mRNA and protein levels by qRT-PCR and western blot, respectively. *P < 0.05.

Next, we observed whether, if so, how ATXN8OS modulated VASP expression. As expected, introduction of si-ATXN8OS, but not a si-NC control, triggered a significant inhibition in VASP expression at both mRNA and protein levels in MCF-7 (Figure 7a and b) and BT-549 (Figure 7c and d) cells. More interestingly, this effect of ATXN8OS knockdown was remarkably abated by restored miR-16-5p expression in the two BC cells (Figure 7a–d). All these results strongly pointed a role of ATXN8OS as a molecular sponge of miR-16-5p to modulate VASP expression.

In addition, we asked whether ATXN8OS modulated cell autophagy by VASP. In contrast to the control group, the transfection of VASP overexpression plasmid significantly reversed the reduction of ATXN8OS knockdown on Beclin 1 expression and LC3 II/I ratio (Supplement Figure S2a and b), showing the repression of ATXN8OS knockdown on cell autophagy by downregulating VASP.

### 3.8 ATXN8OS knockdown enhanced TAMR sensitivity *in vivo*

**Figure 8:**
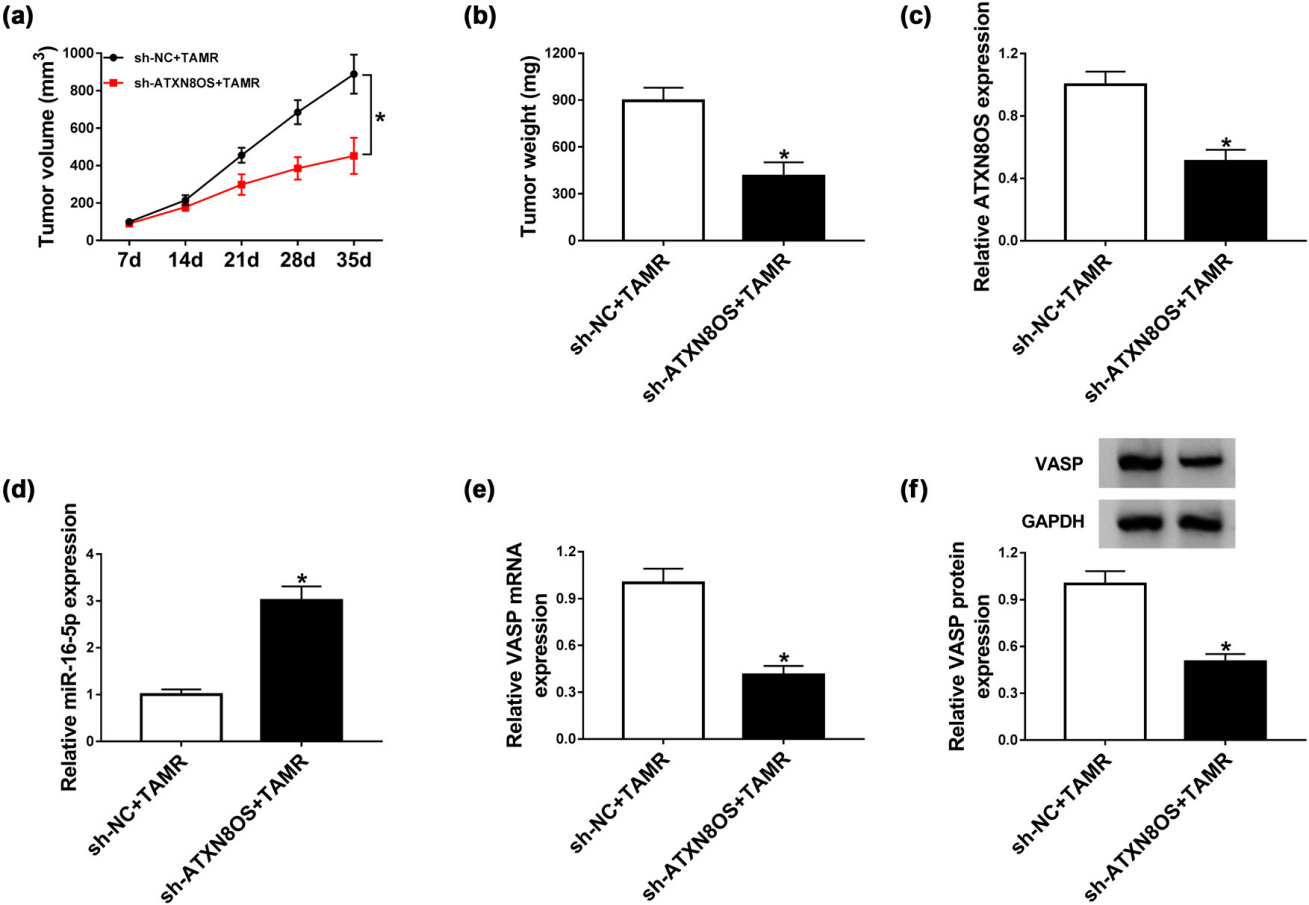
The knockdown of ATXN8OS promoted TAMR sensitivity *in vivo*. MCF-7 cells stably transduced with sh-NC or sh-ATXN8OS were subcutaneously inoculated into the left flank of nude mice, followed by the administration of TAMR (50 mg/kg) every 5 days after 5 days of implantation (*n* = 9 each group). Thirty-five days after injection, all mice were killed to remove the tumor tissues. (a) Tumor volume was assessed every 1 week after first injection. (b) Tumor weight was weighed. The expression levels of ATXN8OS (c), miR-16-5p (d), and VASP (e and f) were determined in xenograft tissues by qRT-PCR and western blot. *P < 0.05.

Finally, we further explored the influence of ATXN8OS knockdown on TAMR sensitivity *in vivo* using xenograft mice model. In contrast to negative control, sh-ATXN8OS transduction promoted TAMR-induced anti-tumor effect, as evidenced by the decrease in tumor volume (Figure 8a) and weight (Figure 8b). As shown by qRT-PCR, ATXN8OS expression was significantly reduced in tumor tissues derived from sh-ATXN8OS-transduced MCF-7 cells (Figure 8c). Moreover, ATXN8OS knockdown resulted in a distinct increase in miR-16-5p expression (Figure 8d) and an obvious decrease in VASP level (Figure 8e and f).

## 4 Discussion

TAMR resistance is still an obstacle in the management of BC, leading to chemotherapy failure. Thus, identifying novel therapeutic targets is very imperative for improving BC treatment. LncRNAs have recently been found to have relevance to the development of TAMR resistance in BC, offering a possibility of lncRNAs as biomarkers for therapy and prognosis of chemoresistant BC [4,21]. In the present study, we first showed that ATXN8OS depletion promoted TAMR sensitivity in BC *in vitro* and *in vivo* partially through sponging miR-16-5p and modulating VASP expression.

In the current work, our data showed a significant upregulation of ATXN8OS in BC tissues and cells, consistent with a previous report [11]. Considering the oncogenic effect of ATXN8OS in BC [11], our study started from the hypothesis that ATXN8OS accelerated BC resistance to TAMR. To confirm this, loss-of-function experiments were carried out using si-ATXN8OS before TAMR treatment, followed by the determination of IC_50_ value, cell apoptosis, and autophagy. Bcl-2 and Bax levels are closely related to the apoptosis in tumor cells [22]. The LC3 II/I ratio and Beclin 1 level are widely acknowledged to reflect the activity of autophagy [23]. In this study, we were the first to uncover that ATXN8OS knockdown augmented TAMR sensitivity in BC cells through enhancing apoptosis and repressing autophagy. Similar to our findings, Wang et al. reported that lncRNA H9 augmented BC cell resistance to TAMR by inducing autophagy [24]. Li et al. discovered that the silencing of lncRNA regulator of reprogramming (ROR) regulated autophagy to abate TAMR resistance in BC cells [25].

It is widely acknowledged that lncRNAs exert their biological functions through acting as a molecular sponge of miRNAs. Therefore, starBase 3.0 software was used to help identify the endogenous interacted miRNAs of ATXN8OS. Among these predicted candidates, miR-16-5p was of interest in the present study owing to its tumor suppressive role in many human cancers, such as malignant mesothelioma, papillary thyroid carcinoma, and hepatocellular carcinoma [26,27,28]. Moreover, Zhang et al. reported that circulating miR-16-5p could act as a potential biomarker for progression indication in gastric cancer [29]. A previous study uncovered that miR-16-5p was a stably expressed housekeeping miRNA in BC tissues from primary tumor to metastatic sites [30]. Subsequently, we first verified that ATXN8OS sequestered miR-16-5p through acting as a miR-16-5p sponge. Our data also showed a significant downregulation of miR-16-5p in BC tissues and cells, in line with earlier researches [13,14]. We further uncovered that miR-16-5p overexpression promoted TAMR sensitivity in BC cells. Previous reports had discovered that several other miRNAs, such as miR-320a and miR-873, could sensitize TAMR-resistant BC cell to TAMR [31,32]. In this study, we also substantiated that the promotional effect of ATXN8OS knockdown on TAMR sensitivity was abolished by restored expression of miR-16-5p in BC cells. In other words, ATXN8OS knockdown enhanced TAMR sensitivity by upregulating miR-16-5p. Deng et al. underscored that ATXN8OS facilitated BC progression through acting as a molecular sponge of miR-204 [11].

Then, we used starBase 3.0 software to help identify the molecular targets of miR-16-5p in humans. Among these candidates, VASP was selected for further research because of its promotional effect on BC cell migration and metastasis [18,33,34]. In addition, the phosphorylation of VASP was reported to drive tumor cell migration and platinum resistance in ovarian cancer [35]. VASP was involved in the regulatory network of miRNA–mRNA to contribute to the development of gemcitabine resistance in human pancreatic cancer cells [36]. In the present study, we were the first to confirm that VASP was directly targeted and repressed by miR-16-5p. Our data also showed that VASP mediated the promotional effect of miR-16-5p overexpression on TAMR sensitivity in BC cells. Moreover, our study first suggested that ATXN8OS modulated VASP expression through acting as a ceRNA of miR-16-5p.

Finally, the xenograft model assay showed that ATXN8OS knockdown enhanced TAMR sensitivity *in vivo*. Moreover, ATXN8OS knockdown triggered an increase in miR-16-5p expression and a decrease in VASP level, eliciting that the knockdown of ATXN8OS augmented BC TAMR sensitivity *in vivo* possibly through miR-16-5p/VASP axis. Therefore, more researches about the role of VASP and the regulatory relationship between ATXN8OS/miR-16-5p axis and VASP on BC TAMR resistance will be performed in further work.

## 5 Conclusion

In conclusion, our study suggested that ATXN8OS knockdown promoted TAMR sensitivity in BC cells *in vitro* and *in vivo* at least partially through miR-16-5p/VASP axis. Our findings highlighted a novel promising therapeutic target for improving the clinical benefits of TAMR treatment in BC patients.
